# CFD Modelling of Abdominal Aortic Aneurysm on Hemodynamic Loads Using a Realistic Geometry with CT

**DOI:** 10.1155/2013/472564

**Published:** 2013-06-24

**Authors:** Eduardo Soudah, E. Y. K. Ng, T. H. Loong, Maurizio Bordone, Uei Pua, Sriram Narayanan

**Affiliations:** ^1^Centre Internacional de Mètodes Numèrics en Enginyeria, Biomedical Engineering Department, Technical University of Catalonia, C/Gran Capità, s/n, 08034 Barcelona, Spain; ^2^School of Mechanical and Aerospace Engineering, Nanyang Technological University, 50 Nanyang Avenue, Singapore 639798; ^3^Department of General Surgery, Tan Tock Seng Hospital, 11 Jalan Tan Tock Seng, Singapore 308433

## Abstract

The objective of this study is to find a correlation between the abdominal aortic aneurysm (AAA) geometric parameters, wall stress shear (WSS), abdominal flow patterns, intraluminal thrombus (ILT), and AAA arterial wall rupture using computational fluid dynamics (CFD). Real AAA 3D models were created by three-dimensional (3D) reconstruction of in vivo acquired computed tomography (CT) images from 5 patients. Based on 3D AAA models, high quality volume meshes were created using an optimal tetrahedral aspect ratio for the whole domain. In order to quantify the WSS and the recirculation inside the AAA, a 3D CFD using finite elements analysis was used. The CFD computation was performed assuming that the arterial wall is rigid and the blood is considered a homogeneous Newtonian fluid with a density of 1050 kg/m^3^ and a kinematic viscosity of 4 × 10^−3^ Pa·s. Parallelization procedures were used in order to increase the performance of the CFD calculations. A relation between AAA geometric parameters (asymmetry index (**β**), saccular index (**γ**), deformation diameter ratio (**χ**), and tortuosity index (**ε**)) and hemodynamic loads was observed, and it could be used as a potential predictor of AAA arterial wall rupture and potential ILT formation.

## 1. Introduction

Most of abdominal aortic aneurysms (AAA) (about 90%) are located below the level of the renal arteries and are known as infrarenal aortic aneurysms. Infrarenal aortic aneurysm is a pathological enlargement of the aorta in the inferior thoracic area taking a fusiform shape and may extend into the iliac arteries. The mortality of this pathology is high (15% for ruptured aneurysms), and the current standard of determining rupture risk is based on the anteroposterior diameter. It is known that smaller AAAs that fall below the threshold of 5.5 cm diameter may also rupture, and yet larger AAAs (diameter > 5.5 cm) may remain stable [1–3]. However, occasionally the AAA diameter is lower than 5 cm and an unexpected ruptured is produced [4], in these cases, other biomechanical factors [5–8], such as Wall Shear Stress (WSS), or geometrical factors [9–12] can play an important role in the rupture of the aneurysm. 

To estimate the AAA rupture risk, from a biomechanical point of view (material failure), an aneurysm ruptures when the stresses acting on the arterial wall exceed its failure strength. According to Laplace's law, the wall stress on an ideal cylinder is directly proportional to its radius and intraluminal pressure. Even though an AAA is not an ideal cylinder, Laplace's law still applies and with an increasing aortic diameter, the internal pressure increases, and so does the risk of rupture. The increase in internal pressure against the aortic walls results in progressive growth of the AAA diameter, and, eventually, this pressure may overcome the resistance of the aortic wall resulting in rupture [1, 2]. On the other hand, abnormal flow patterns and recirculation develop in the AAA sac leading to the formation of the intraluminal thrombus (ILT) [6, 13]. This phenomenon can provoke the AAA stabilization and start a vicious circle inside the AAA [14]. It is reflected by the interaction between the arterial wall structural remodeling and the forces generated by blood flow within the AAA [2, 13]. Therefore, it is apparent that Laplace's Law is insufficient when investigating AAA collapsibility. Rather, the aneurysm shape has a strong influence on flow patterns, ILT formation, wall stress distribution (peak values and locations), and consequently its potential rupture [1, 15].

The aim of this study is to analyze and to characterize the effect of wall shear stress and the internal pressure together with the main AAA geometric parameters (maximum diameter (*D*_AMAX_), length (*L*_AAA_), AAA proximal neck diameter (*d*_proximal_neck_), tortuosity (*ε*), and asymmetry (*β*)) in order to assess its potential rupture. Five patient-specific AAA models were created from CT scans. A normal descending aorta was also simulated to provide a comparison.

## 2. Material and Methods

Five patients with infrarenal aneurysms on followup at Tan Tock Seng Hospital (Singapore) were included in this study. The patients chosen for this study were selected with different sized AAAs, in order to cover the different stages of this pathology. All the patients participated in this trial analysis volunteered and provided written informed consent of the study. This study was reviewed and approved by the Ethics Committee of the Tan Tock Seng Hospital, Singapore. For the medical image acquisition, a computed tomography (CT) Somatom Plus Scanner (*AS+*) (Siemens Medical Solutions) was used with the following parameters: 512 × 512 × 110, pixel spacing: 0.785/0.785 with a resolution of 1.274 pixels per mm and 5 mm slice thickness. CT scanning was conducted while the volunteer was awake in the head first-supine position using an endoleak protocol. The CT covered the entire abdomen and pelvis and was performed after the administration of intravenous Omnipaque 350 as IV contrast medium.

**Figure 1:**
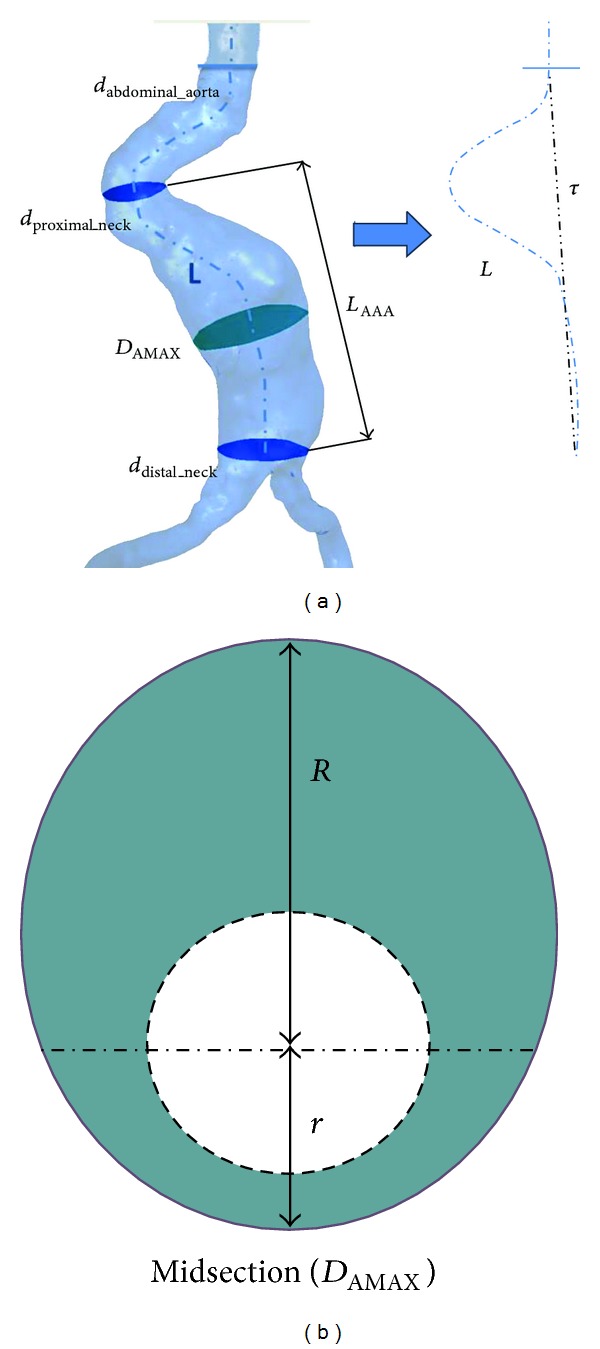
(a) Main geometrical parameters: *L*_AAA_ aneurysm length, *D*_MAX_ maximum diameter of the aneurysm *d*_proximal_neck_ beginning of the AAA sac, *d*_distal_neck_ ending of the AAA sac, *d*_abdominal_aorta_ nondeformed abdominal aorta diameter, *L* is the absolute length of the tortuous vessel, and *τ* is the imaginary straight line. (b) Schematic visualization of a cross-sectional AAA section, where *r* and *R* are defined as the radii measured at the midsection of the AAA sac from the longitudinal *z*-axis to the posterior and anterior walls.

To characterize the structure of the AAA, the main geometrical AAA parameters are measured: aneurysm length (*L*_AAA_) and maximum diameter of the aneurysm (*D*_AMAX_) (Figure 1(a)). The factor which assesses the length (*L*_AAA_) and the diameter (*D*_AMAX_) of the AAA sac is known as saccular index (*γ*) (1) [16]. If the saccular index is close to 1, the aneurysm is saccular (spherical), but if it is close to 0 the aneurysm is more fusiform. The deformation diameter rate (*χ*) (1) [17] characterizes the nondeformed abdominal aorta diameter (*d*_proximal_neck_) with the maximum diameter of the aneurysm sac, *D*_AMAX_. A nonaneurysmal aorta is defined as (*D*_AMAX_ = *d*_neck_)

(1) $$\begin{array}{c} \beta =\frac{r}{R},\\ \gamma =\frac{D_{\text{AMAX}}}{L_{\text{AAA}}},\\ \chi =\frac{D_{\text{AMAX}}}{d_{\text{proximal}\_\text{neck}}},\\ \varepsilon =\;\frac{L}{\tau }-1. \end{array}$$

To evaluate the asymmetry (*β*) [18] of the aneurysm (1), *r* and *R* are defined as the radii measured at the midsection of the AAA sac from the longitudinal *z*-axis to the posterior and anterior walls, respectively, as shown in the inset of Figure 1(b). Thus, *β* = 1.0 yields an azimuthal symmetry and *β* = 0.2 is an AAA for which only the anterior wall is dilated while the posterior wall is nearly flat. The tortuosity index (*ε*) (1) [19] is the relation between the actual lengths of the centerline of the AAA with the length of a hypothetical healthy aorta (Figure 1, center).

Based on these indexes and the wide clinical empirical evidence, there are several criteria of the AAA grade. However, at present, there is no clinical consensus to use it.  Table 1 shows the main AAA geometrical characteristics for each patient in our study. 

**Figure 2:**
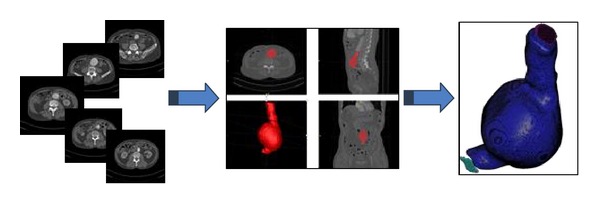
Workflow of the 3D AAA model. Obtaining the CT image of the abdominal aorta, segmentation of the vessel lumen using thresholding and level set method, and 3D model of the AAA.

To create the computational model, the medical data were sent directly to a personal computer and stored in Digital Imaging and COmmunications in Medicine format (DICOM format).  Figure 2 shows the segmentation workflow. The region of interest (ROI) analyzed was segmented using the three-dimensional computer-aided design system DIPPO software [20]. The segmented area for each patient started at the abdominal aorta (approximately in the infra renal arteries) and extended down to the common iliac arteries (Figure 2). The abdominal images were segmented from CT DICOM images combining two different segmentation procedures, thresholding and level set method (based on snakes). Thresholding is a nonlinear operation that converts a gray-scale image into a binary image where the two levels are assigned to pixels that are below or above the specified threshold value. The image snake operation creates or modifies an active contour/snake in a greyscale image. The operation iterates to minimize the snake's energy which consists of multiple components including the length of the snake, its curvature, and image gradient [21].

After AAA segmentation, we get a 3D volume image useful to create a 3D computational model to analyze the blood flow behavior inside the AAA using computational fluid dynamics (CFD). A mesh sensitivity analysis was performed to ensure the accuracy of the simulations using steady test. Depending on the complexity of the AAA model, a 3D mesh consisted of 2.000.000–2.500.000 tetrahedral elements. Using the isosurface stuffing algorithm [22], we have obtained a smooth element and an aspect radio for the whole of the meshes higher than 0.9 (ideal ratio = 1 for an equilateral triangle). For the five acquisitions, the same medical image protocol, image processing, and volume mesh reconstruction were used.

## 3. Computational Fluid Mechanics Solver

CFD analysis was performed using BioDyn, a friendly user interface based on the commercial software Tdyn [23]. Tdyn is a fluid dynamics and multiphysics simulation environment based on the stabilized finite element method that solved the Navier-Stokes equations. To characterize accurately the blood flow in the AAA, a Reynolds number was calculated for whole cases. Reynolds number is a dimensionless number that determinates the dynamic of the fluid. Reynolds number is defined as *Re* = *UD*/*ν*, where *U* is the mean velocity, *ν* is the kinematic viscosity of air, and *D* is the characteristic length given as the hydraulic diameter *D* = 4*A*/*P* for the inlet velocity, and here *A* is the cross-sectional area and *P* is the perimeter of the aorta. Because the Reynolds number in inlet is low (<1000), we decided to use a CFD solver for laminar flow considering steady, homogeneous, incompressible, adiabatic, and Newtonian fluid. However, three-dimensional flow features such as flow separation and recirculation might trigger a transition to turbulence at lower Reynolds numbers [9]. Based on a preliminary study [6], the effect of the turbulence has been considered to be negligible. Following, we show the Navier-Stokes equations (2)

**Figure 3:**
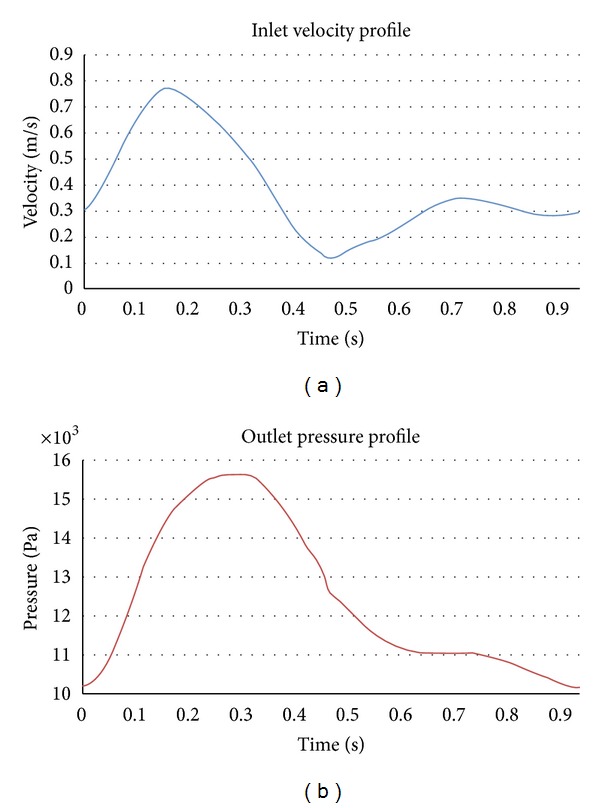
Boundary conditions for the hemodynamic simulations, adapted from [11].

**Figure 4:**
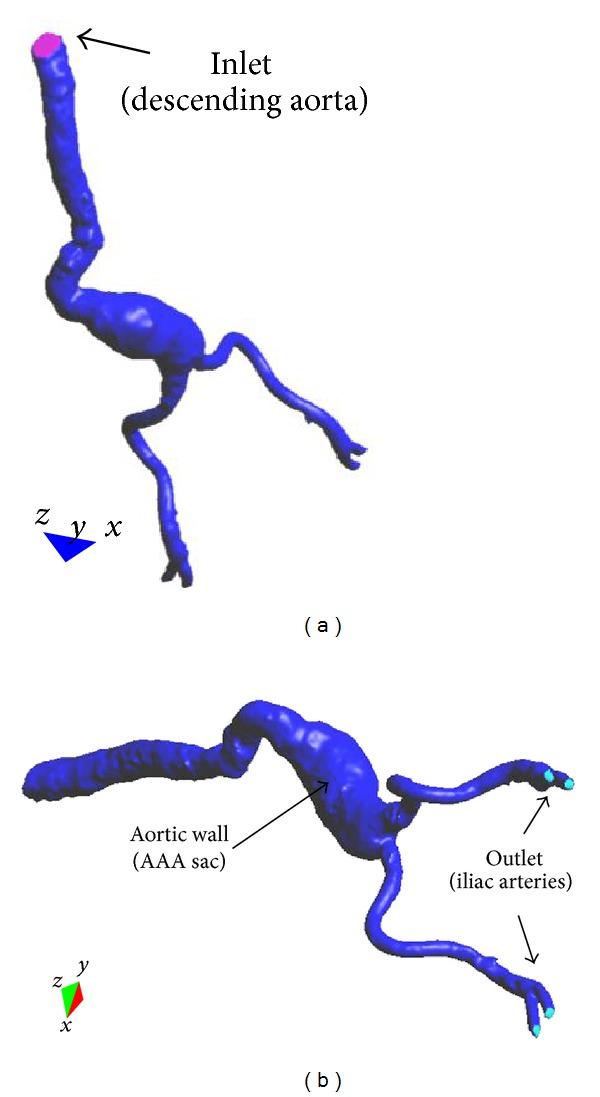
Mesh surface: different layers are shown: aortic wall (dark blue) and inlet (purple) and outlet sections (light blue).

(2) ρ(∂u∂t+(u·∇)u)+∇p−∇·(μ∇u)=ρf, in  Ω×(0,t),∇·u=0,   in  Ω×(0,t),

where *u* = *u*(*x*, *t*) denotes the velocity vector, *p* = *p*(*x*, *t*) the pressure field, and the density (*ρ*) is considered constant with a value of 1050 Kg·m^−3^ and dynamic viscosity (*ν*), fixed at 0.004 Pa·s and *f* the volumetric acceleration. The spatial discretization of the Navier-Stokes equations has been done by means of the finite element method (FEM), while for the time discretization an iterative algorithm that can be considered as an implicit two steps fractional step method has been used. A new stabilisation method, known as finite increment calculus, has recently been developed [24]. By considering the balance of flux over a finite sized domain, higher order terms naturally appear in the governing equations, which supply the necessary stability for a classical Galerkin finite element discretization to be used with equal order velocity and pressure interpolations. The inlet velocity waveform was taken from the literature [25], and Figure 3 shows the pulsatile waveforms used. For inlet condition, a transient blood flow was imposed in the descendent abdominal aorta. The velocity *U* was calculated for each patient in order to obtain a total fluid volumetric flow rate of 500 mL for an entire cardiac cycle. The outlet boundaries were located at the common iliac arteries where the pressure follows pulsatile waveforms as defined in Figure 4(b). It is important to remark that these profiles are not patient-specific data (MR velocity mapping was not performed on these subjects), which can be a limitation of this study. In further studies, a patient-specific velocity and pressure profile will be used as boundary conditions.

Mathematically, the boundary conditions can be expressed as in (3a), (3b), and (3c). No-slip condition (vessel rigid wall) was imposed on the surface of the arteries, (3a). This choice is motivated by the fact that the physiological parameters characterizing the arterial mechanical behaviour of the AAA wall are not well determined. This approach, however, reduces the discretization effort considerably, in particular boundary layer gridding and the computational cost although other approaches consider fluid structure interaction (FSI) models [13, 25–28]. The inlet velocity is assumed to fully developed their parabolic profile at the inlet (3b), and time-dependent normal traction due to luminal pressure at the outlet (3c) as

(3a) $$\begin{array}{c} V=0|{}_{\text{wall}}, \end{array}$$

(3b) $$\begin{array}{c} u_{z}=2\left(u\left(t\right)\right)\left(1-{\left(\frac{2r}{\;d_{r}\;}\right)}^{2}\right);\;\;{u_{r}=0|}_{z=0}, \end{array}$$

(3c) $$\begin{array}{c} \tau_{nn}=\hat{n}\cdot p\left(t\right)I\cdot \hat{n}, \end{array}$$

where *d*_*r*_ is the inner radius of the abdominal aorta, *u*_*r*_ is the Cartesian component of the velocity vector in the “*z*” direction, and *u*(*t*) and *p*(*t*) are the time-dependent velocity and pressure waveforms designated in Figure 3. The pressure boundary conditions are given by (3c), where *τ*_*nn*_ is the normal traction designates at the outlet, *I* is the standard identity matrix, and $\hat{n}$ designates the normal of the respective boundary.  Figure 4 shows case 4 of the AAA reconstructed model and the layers in which our domain is divided in order to impose the boundary conditions. 

## 4. Results

Five models of infrarenal aneurysm with patient-specific geometry were analyzed using computational fluid dynamics in order to evaluate the flow patterns, wall shear stress, and pressure over the aneurysm sac. Patients 1 to 4 had infrarenal aneurysms whilst patient 5 is the control case. ILT was not found in any case. The unsteady flow simulations were performed through two pulsatile cycles to eliminate the influence of initial transients. The results of pressure, stress, and flow patterns are shown for the peak systole (0.2 s) of the second cardiac cycle.

**Figure 5:**
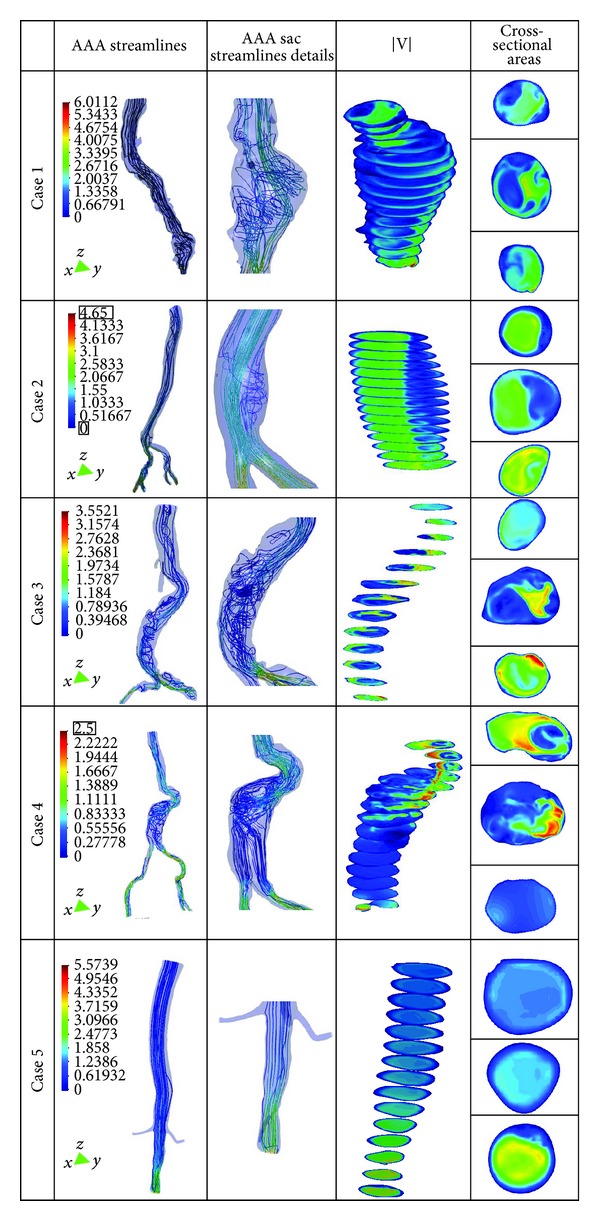
Streamlines, details of AAA sac streamline, blood flow velocity, and three cross-sectional areas (from the top to the bottom: proximal AAA neck and midsection and distal AAA neck) for the five patient studied.

Figure 5 shows the flow patterns, velocity streamlines inside the infrarenal aneurysms sac, and three cross-sectional areas of the AAA (proximal, midsection, and distal neck) for the five patients studied. Note that forward flow points downwards and has a negative value in the adopted coordinate system. The direction of the flow is from top to bottom (direction *Z* negative). We notice that when the AAA has asymmetry, the velocity streamlines correspondingly show an asymmetric flow pattern inside the AAA sac. As shown in [6, 7, 26, 29], the asymmetric flow patterns can provide an insight into the mechanism that promotes the thrombus renewal and possibly enlargement inside the aneurysm. Rapid decrease in the velocity and regions of very high (or low) hemodynamic stresses gradients may all contribute in various ways to the vascular disease, primarily via their effects on the endothelium. For example, platelets trapped in recirculation zones tend to be deposited in areas of low shear stress (stenosis) [14], since this and the presence of vortices cause prolonged contact of the platelets with the surface in the layer of slow fluid motion [6, 19]. This effect can be observed for the pathological cases; however, in case 4 we observe a flow recirculation in the middle section and the asymmetry is low. Consequently, the flow patterns of the aneurysm not only depend of the asymmetry of the aneurysm but also depend of the AAA tortuosity. Details of the flow patterns for the AAAs is shown in Figure 5 (cross-sectional areas column). From the top to the bottom: neck section, midsection (maximum diameter in the aneurysm sac), and distal section. We notice that the tortuosity of the AAA provokes an asymmetric flow patterns in the AAA sac. When *ε* is high, we observe that in the AAA proximal neck section there is a strong irregular flow, causing also an irregular flow in the AAA sac meanwhile, when *ε* is low, this effect does not appear. From these findings, the greater the asymmetry and tortuosity of the AAA, the higher the possibility of blood recirculation, ILT formation, and a possible rupture. Thus, the importance of geometry in the hemodynamic behavior of AAAs is supported.

**Figure 6:**
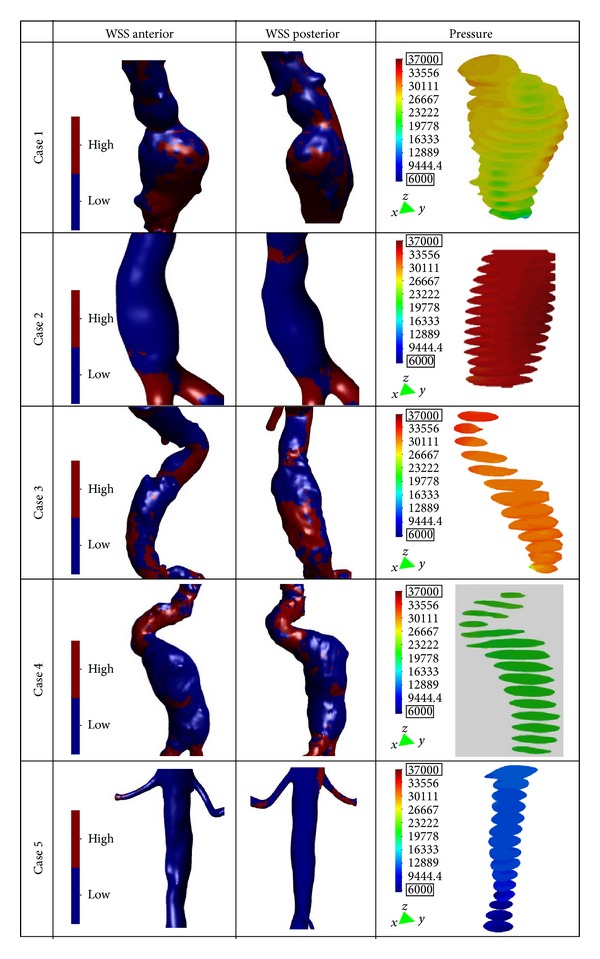
Anterior and posterior wall shear stress views (high WSS in red, and low WSS in blue) and normalized pressure on the aneurysm sac at the peak systole for the five patients.

Anterior and posterior wall shear stress views are shown in Figure 6, as well as the pressure on the aneurysm sac at the peak systole. The mean WSS in the aneurysm sac of the 5 patients varied between 0.28 (case 5) and 12.72 Pa (case 4) with a median of 3.52 Pa and mean of 5.74 Pa. The areas with WSS values under the mean value have been classified as low WSS areas (blue) and areas with WSS values higher than the mean value as high WSS (red). Analysis of WSS maps for all cases shows that the area of low WSS coincides with the location of the recirculation areas (low velocities), and higher WSS values are found in two regions: in the neck area and in the corresponding area where the blood flow jet has an impact on the aneurismal sac. The blood flow jet has an impact directly on the arterial wall producing higher WSS values. The blood flow jet path is provoked by the proximal neck angle, consequently by the AAA tortuosity (see Figure 5). These WSS peak values do not influence too much in aneurysm growth; however, in these areas a material failure could be produced provoked by blood flow jet. It is interesting to note that shear stress levels in all infrarenal aneurysms models are higher than those in the normal aorta, where *ε* is low, which has a fairly uniform stress shear spatial distribution. Maximal values are found in the neck region, where *ε* is high.

Due to the increasing of the internal pressure, against the aortic walls, the AAA diameter grows progressively, and eventually it could be able to overcome the resistance of the aortic wall with its breakup. This internal pressure is directly proportional with the aneurysm diameter; thereby, when the diameter grows, the pressure increases. High deformation diameter rate (*χ*) index could indicate higher internal pressure however, it should be noticed that the pressure also depends on the tortuosity index (inversely proportional) and the flow characteristics (see Figure 5). For instance, in case 4 even when the section diameter is large, the internal pressure is lower than that in case 2. This is originated by a notably pressure drop as a result of the vorticity and flow recirculation (energy losses) in the cases with a high tortuosity index. This effect also provokes a pressures gradient between the anterior and posterior arterial wall in the interior of the AAA sac. These internal high pressure areas inside the sac may indicate the growth directions of AAA sac.

## 5. Discussions

The objective of this study is to use patient-specific AAA models for correlating the geometric indices of the aneurysm with the hemodynamic loads and, eventually, with the potential risk of AAA rupture. From a mechanical point of view, AAA ruptures when a maximum stress value over the wall is exceeded. The stress peak refers to the mechanical load sustained by the AAA wall, during maximal systolic pressurization. In addition, it is known that wall stress alone does not completely govern failure as an AAA will usually rupture when the wall stress exceeds the wall strength. Its value depends on arterial systolic pressure, the mechanical properties, and the geometric configuration of the material under study. However, the mechanism of arterial wall failure due to (1) the AAA shape or (2) the pressure distribution is physically different. The first is because of a punctual force over a wall point, and the second is because a consequence of a pressure distribution over the AAA wall. Based on the AAA shape, several authors have proposed different criteria for the AAA collapsibility; if the AAA diameter is higher than 5.5 cm [4], the AAA may rupture or if the asymmetry index factor (*β*) is *β* < 0.4, the AAA rupture is high [8]. In [10] the AAA collapses when the deformation diameter ratio is *χ* > 3.3 or if the saccular index is <0.6 [11]. But depending on the index that is analysed, the surgical criteria are different. More recently, the proposed AAA rupture risk is related with the presence of intraluminal thrombus (ILT) [6, 26]. The intraluminal thrombus plays an important role in expansion and rupture of advanced aneurysms through direct mechanical as well as indirect chemomechanical effects [6]. Therefore, the AAA dilatation (and a potential rupture) is dependent on the correlation between the geometrical factor and the hemodynamic load. Consequently, alternative methods of AAA rupture assessment are needed.

The majority of these new approaches involve the numerical analysis of AAAs using computational fluid dynamics (CFD) to determine the wall shear stress (WSS) distributions and flow patterns in the aneurysm sac [5, 12, 13, 15, 16, 30]. In our study, we did not evaluate the patient-specific wall strength or the effect of intraluminal thrombus. Nevertheless, the AAA cases analyzed provide useful information to understanding how hemodynamic loads can affect aneurysm growth and possible rupture. The pathological cases (cases 1 to 4) always present irregular flow in the aneurysm sac, no uniform distribution of WSSs and great WSS values in the curvatures of the aortic vessel. These anomalies are proportional to the shape of the aneurysm and the angles of twist that the aorta has. Asymmetric flow is always correlated to the modified curvature of the vessel or when some enlargement in the aneurysm is present (high saccular index (*γ*) and tortuosity (*ε*) index). The AAA tortuosity could initiate ILT formation and at the same time could provoke arterial wall failure. In a situation where the tortuosity is high and the saccular index is low, the WSS has more effects than the pressure distribution in a possible AAA failure. However, where the AAA is not twisted and the saccular index is high, the pressure effect takes priority. In Figure 6, the highest shear stress values are dependent especially on the tortuosity of the AAA. The neck angle substantially impacts flow fields, causing strong irregular flow patterns in the AAA sac (Figure 5) significantly influencing wall stress distribution (Figure 6). The relationship between these indexes can provide an insight into the mechanism that promotes the thrombus renewal and possibly enlargement inside the aneurysm. 

## 6. Conclusions

To conclude, in this work, the wall shear stress, internal pressure, and flow patterns of five patients-specific aortas have been analyzed using a finite element method, and the results have been correlated with the geometrical features. Results from the patient-specific infrarenal aneurysm models (cases 1–4) were compared with those of a normal aorta (case 5) and it has been found that the normal aorta has a uniform distribution of the velocity streamlines, wall shear stress, and pressure compared to that in the pathological cases. Maximum wall shear stresses in all infrarenal aneurysm models are higher than those in the normal aorta and these values are not directly related to the maximum aneurysm diameter, providing evidence that a patient-specific aorta shape analysis is necessary for a more reliable assessment of the rupture risk of aortic aneurysms. Therefore, based on one or two simple indexes alone, to determine the risk of rupture accurately is insufficient. AAA rupture is a complex situation, depending on the maximum diameter, internal pressure, wall stress, asymmetry, saccular index, intraluminal thrombus, and tortuosity, among others. Our results demonstrate how the hemodynamic loads as simulated by computational fluid dynamics (CFD) are influenced by the geometrical factors of the aneurysm. 

## Figures and Tables

**Table 1:** Patient and AAA geometrical characteristics (*L*, τ, *d*_proximal_neck_, *r*, *R*, *D*_AMAX_, and *L*_AAA_), and all the measurements are expressed in centimeter. AAA geometrical indexes (*β*, *γ*, *χ*, and *ε*).

Case	Sex	Thoracic aorta length (*L*)	Hypothetic thoracic aorta length (*τ*)	AAA proximal neck (*d*_proximal_neck_)	*r*	*R*	AAA max diameter (*D*_AMAX_)	AAA length (*L*_AAA_)	*χ*	*β*	*γ*	*ε*
1	Male	359,94	320,44	2,8	1,49	2,45	3,945	6,7	1,408	0,608	0,588	0,123
2	Male	319,5	310,2	1,7	0,67	1,74	2,416	4,1	1,421	0,388	0,589	0,029
3	Male	216,28	176,8	2,6	1,02	2,03	3,056	8,98	1,176	0,508	0,340	0,223
4	Male	293,55	255,26	2,6	0,95	3,07	4,031	15,6	1,55	0,309	0,258	0,150
5	Male	310,34	296,24	2,5	1,25	1,25	2,5	—	1	1	—	0,047
